# Kalman Filter Based Load Monitoring in Beam Like Structures Using Fibre-Optic Strain Sensors

**DOI:** 10.3390/s19010103

**Published:** 2018-12-29

**Authors:** Rohan Soman, Wieslaw Ostachowicz

**Affiliations:** Institute of Fluid Flow Machinery, Polish Academy of Sciences, Gdansk 80-231, Poland; wieslaw@imp.gda.pl

**Keywords:** structural health monitoring, fibre optic sensors, load monitoring, strain

## Abstract

The paper presents a proof of concept of a new methodology for the load estimation in beam-like structures under complex loading. The paper customizes a Kalman Filter (KF) based estimation technique which is shown to be robust to the presence of measurement noise as well as the changing condition of the beam for estimation of loads in beam-like structures. The methodology was validated using numerical as well as experimental data. The initial studies indicate that the proposed methodology has promise for applications where monitoring and classification of the strains is necessary, such as those in continuous welded rails.

## 1. Introduction

Structural health monitoring (SHM) is no longer a luxury but a necessity. The safety and reliability of structures is of utmost importance in the present day due to the heavy dependence of human activities on these structures. A collapse of a structure may have huge financial impact and may even cause loss of life. To ensure reliable functioning of structures, they are instrumented with different kinds of sensors for measurement of the in-service performance. The use of strain sensors is very common and in the last decade fiber optic sensors are being extensively used. The benefits offered by the fiber optics can be summarized as [1]:

(i) Low power loss
(ii) Resistance to electromagnetic interference
(iii) Low security risk
(iv) Small size
(v) Lightweight
(vi) Easy to accommodate increasing bandwidth
(vii) Resistant to harsh conditions

In most structures, in addition to the monitoring of the performance, monitoring of the loads is also critical. The load monitoring and the response then can be used to develop guideline for the safe use of the infrastructure, especially after an extreme event such as an earthquake.

The loading on the structure can be classified based on the way it is applied, including impact load, dynamic load and static load. In addition, the loading can also be classified based on the direction of influence, for example bending load or axial load. In some applications, the nature of the loading is just as important as the magnitude of the load and it is necessary to classify and measure the different types of loads in the structure. One common example is the continuous welded rail (CWR) where the axial compressive loads due to the approaching locomotive are far more important than the bending loads due to the passing locomotive. The resilience of the structure to bending is much higher than that to buckling as a result it is of utmost importance to monitor the axial loads and determine safety limits for the speed of the train. Excessive buckling loads on the tracks may lead to track misalignment and result in an accident. Several attempts have been made for the rail axial load monitoring, using a variety of different sensors including strain gauges and fiber optic (FO) sensors, using distributed sensing as well as fiber Bragg grating (FBG) sensors [2,3,4,5]. The problems with these techniques are the accuracy of the estimation and in the case of the change in the structure, the inability to avoid false errors. In addition, some research work proposes specially designed sensors and hardware for the application [6]. The customization of the sensors and the need for specialized equipment increases the cost. Hence, the proposed paper addresses the issue of specialized sensors by using off the shelf sensors and provides better estimation through the use of smart signal processing. Some works on the use of FO sensors in particular orientation and post processing for temperature compensation have also been carried out [7,8,9,10,11].

In addition to the CWR applications, buckling load monitoring is also equally important in structures that use composite materials. Composites have a very high strength to weight ratio and allow us the freedom of tailoring the properties of the structure to our needs. However, the non-homogenous material and the difference in the mechanical properties of the constituents of the composite leave it highly susceptible to buckling. Hence, there is a need to monitor the axial and bending loads in the structure. The composites in addition to the incomparable mechanical properties also offer us an advantage where we can embed the sensor within the structure to be monitored. The FO sensors are ideally suited for embedding and may be used without significant detriment to the structure [12].

Some other applications of load monitoring may be found in ice detection on wind turbine blades or transmission lines [13] or in the aerospace industry as well as civil engineering applications. In civil engineering applications, the load monitoring is quite essential for the prestress force monitoring in prestress concrete girders [14] or for monitoring of the curing process of concrete as well as the cable force monitoring in cable-stayed bridges [15].

Some other works in the load monitoring of beam structures have been carried out as well [16,17,18]. The most notable of these works were by Ma et al. and Lin et al [19,20,21]. Ma et al. [19,20] proposed the use of Kalman Filter (KF) for the suppression of noise. Building on the work by Ma et al., Lin et al. [21] used the extended KF for linearizing the nonlinear systems and in turn improving the applicability of these methods. However, the work carried out remains largely theoretical as it is difficult to obtain the measurement of nodal displacements and rotations at many locations in real structures. These papers further support the use of KF for the use in load estimation with noisy data. The shortcomings of the need for the nodal information is overcome in the present work through an innovative formulation of the problem and implementation of the KF. The KF remains a highly used technique for estimation and multi-sensor fusion in host of different applications due to its excellent performance under measurement uncertainties [22,23,24].

Thus, this paper proposes a technique for the monitoring of the axial and bending loads on a composite sample using fiber Bragg grating (FBG) sensors. To make the measurements robust to measurement noise, a Kalman filter (KF) based formulation is proposed. For a linear system, the KF gives the least mean square error (best estimate). In addition, the formulation allows the use of the sensors for damage detection while performing the load assessment. The paper presents customizations of the KF based methodology for the application to load estimation. A modified implementation of the measurement update matrix improves the stability and the accuracy of the load estimation as compared to the earlier publications and as such is the novelty of this paper.

The paper is organized as follows. The methodology and the formulation of the system equations are described in Section 2. The Section 2 also describes the KF formulation. The subsequent section, Section 3 describes the experimental setup. The results including the need for the KF based techniques under different conditions for the numerical and experimental study are presented in Section 4 and Section 5, respectively. Based on the observed results some conclusions and the areas for future work as well as possible applications of the methodology proposed are identified in Section 6.

## 2. Theoretical Background

### 2.1. Beam Theory

**Figure 1:**
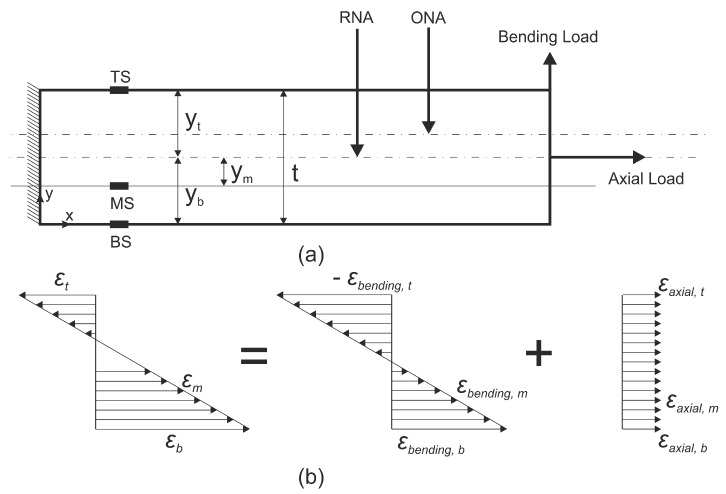
(**a**) Beam under axial and bending loads; and (**b**) superposition of bending and axial strains, where $y_{t}$, $y_{b}$ and $y_{m}$ are distances from the real neutral axis (RNA) to the top, bottom and middle sensor, respectively, t is the sample thickness, and ONA is the observed neutral axis.

Figure 1 shows a beam under combined bending and axial loading (complex loading). The figure also shows the strain profiles due to the individual axial and bending load as well as the superimposed combined distribution. Based on the strain profiles, the strains measured at the three strain sensors resolved into the components are given by Equations (1)–(3)

(1) $$\epsilon_{t}=\epsilon_{axial}+\epsilon_{bending,t}$$

(2) $$\epsilon_{b}=\epsilon_{axial}+\epsilon_{bending,b}$$

(3) $$\epsilon_{m}=\epsilon_{axial}+m\times \epsilon_{bending,b}$$

where *m* is the ratio of the distance of the sensors MS and BS from the real neutral axis (RNA) ($m=\frac{y_{m}}{y_{b}}$); subscripts bending and axial refer to strains in those directions; and subscripts *b* and *t* refer to the strains at different cross sections.

In the application at hand, the aim is to estimate the strains in bending and along the axis. These strains are a function of the applied loads, the condition of the structure and the ambient temperature conditions. There is a need to incorporate the condition of the structure in the estimate of the strains measured. Hence, the state space model is given as:

(4) $${\left[\begin{array}{c} S_{A}\\ S_{B}\\ RNA \end{array}\right]}_{k}=\left[\begin{array}{ccc} 1 & 0 & 0\\ 0 & 1 & 0\\ 0 & 0 & 1 \end{array}\right]{\left[\begin{array}{c} S_{A}\\ S_{B}\\ RNA \end{array}\right]}_{k-1}+w_{k-1}$$

(5) $${\left[\begin{array}{c} \varepsilon_{b}\\ \varepsilon_{m}\\ \varepsilon_{t} \end{array}\right]}_{k}=\left[\begin{array}{ccc} 1-\frac{\alpha }{RNA_{k-1}} & 1 & 0\\ 1 & 1 & 0\\ 1 & 0 & 2\times S_{B_{k-1}} \end{array}\right]{\left[\begin{array}{c} S_{A}\\ S_{B}\\ RNA \end{array}\right]}_{k}+v_{k}$$

where $S_{A}$ is the axial strain estimate, $S_{B}$ is the bending strain estimate, RNA is the real neutral axis location, $w_{k-1}$ is the process noise with zero mean and covariance (Q), α is the ratio of the distance between MS and BS ($\frac{y_{b}-y_{m}}{t}$) and $v_{k}$ is the measurement noise with zero mean and the covariance (R).

### 2.2. Kalman Filter

The KF is a set of mathematical equations that provides an efficient computational (recursive) solution of the least-squares method. KF combines a system’s dynamic model (physical laws of motion) and measurements (sensor readings) to form an estimate of the systems varying quantities (system state) that is better than the estimate of the system obtained by measurement alone [25]. The KF is the theoretical least error estimate of the system when the system is linear, and the uncertainty is Gaussian [26]. Hence, given the linear association between the strain, and the loads as well as the RNA, the KF is an ideal iterative estimation tool. In addition, it is recursive and robust to noise in both the process and the measurement model. In addition, the KF allows easy multi-rate data fusion from different sensor types and is ideally suited for incorporating other information such as temperature or loading conditions into the estimation process.

**Figure 2:**
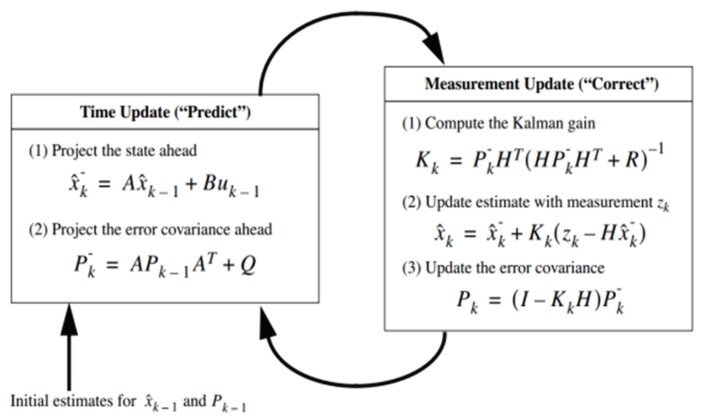
Flow Chart for the implementation of the KF, where *x* is the estimate of the state, *A* is the state transition matrix, *B* is the control matrix, *u* is the control variable, *P* is the state variance matrix, *Q* is the process variance matrix, *K* is the Kalman gain, *H* is the measurement matrix, *z* is the measurement variable, the “super minus” indicates a prior estimate and the subscript *k* indicates the time step.

Based on the state space model, the KF may be implemented. A detailed description of the matrices in the implementation of the KF can be found in Ref. [27]. A concise explanation of the KF is shown in Figure 2.

The key changes to the implementation are in the measurement matrix (*H* in Figure 2), which is significantly different. This change was made as the use of the KF is now for load monitoring and the neutral axis tracking is auxiliary to the main application. In addition, the replacement of the scaling factor with a KF based estimated quantity from the previous iteration results in more stable estimations. This stability comes by compromising on the speed of the convergence of the RNA estimate to the true value but gives more accuracy to the load monitoring and hence is preferred in this particular application.

## 3. Experimental Setup

**Figure 3:**
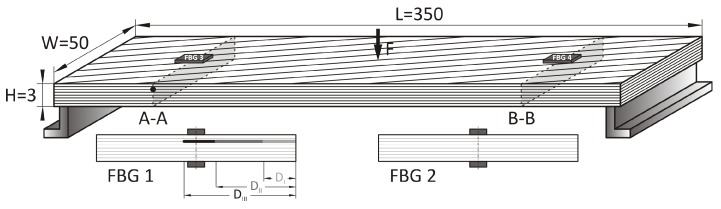
Details of composite specimen with cross-section views at sensor locations.

The composite beam was made out of eight layers of woven fabric of glass fibers (S-glass) with the weave (+45/−45) each of which is 0.2 mm thick. The layers are placed to maintain the symmetry of the composite sample and ensure that the NA will be at the center. The matrix material was Distitron VE 100. The composite beam was then instrumented with two pairs of 1 mm gauge length FBG sensors (Micron optic os1100) (Figure 3).

**Figure 4:**
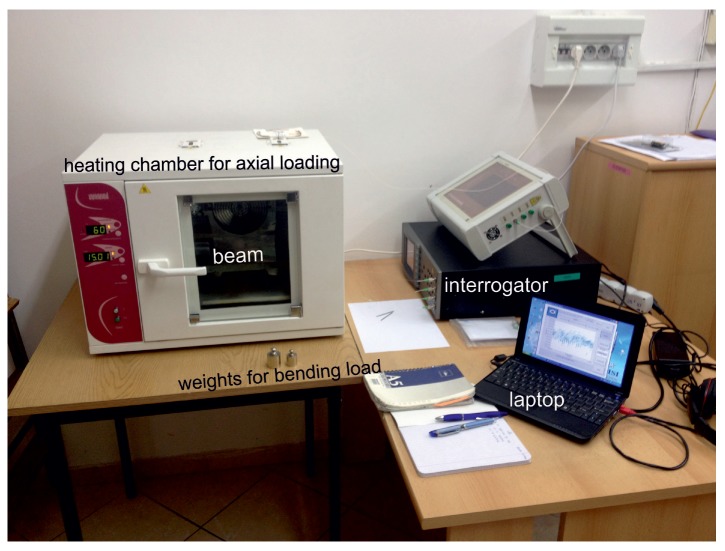
Experimental setup.

The sample was placed in a heating chamber (Wamed Sp. z.o.o) with the operation range from 5 °C to 250 °C, as shown in Figure 4. The chamber was used for changing the axial load on the sample through thermal expansion. Apart from the two sensor pairs, one sensor was multiplexed to measure the temperature inside the chamber. This sensor was used to monitor the axial strain induced in the system due to the the thermal loading. In real structures, this sensor is equivalent to having a sensor placed at the RNA of the beam. This placement ensures that the sensor measures only axial strain and is insensitive to bending loads. This approach was preferred due to the known problems associated with the embedding of the FBG sensors, e.g., splitting of wavelength peak and incorrect bonding with the material [28,29,30].

Due to the limitations of experimental apparatus, the tests were limited to only static bending loads and the axial loads applied based on the thermo-elastic equivalence. For determining the efficacy of the proposed technique under different loading conditions, some validation was carried out on a numerical model of the composite beam. The details of the modeling and validation are provided in Ref. [31]. The results are discussed in the next section.

### Strain Measurements

**Figure 5:**
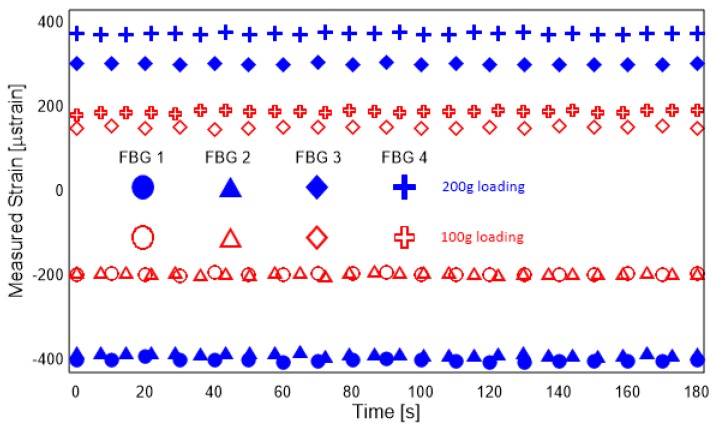
Response of beam under bending loads.

**Figure 6:**
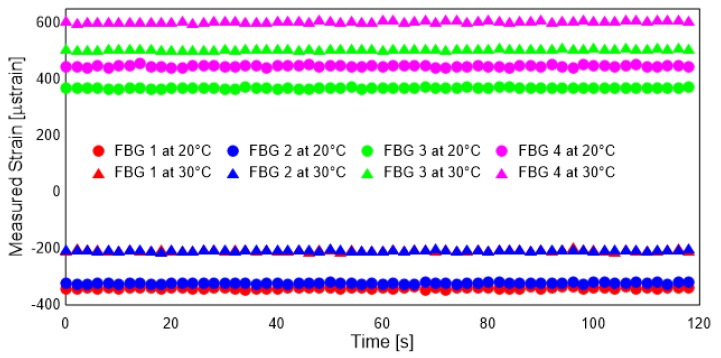
Response of beam under thermal (axial) loads.

The strain measurements were carried out for the beam with the experimental setup explained in the previous section. For the purpose of the study, the beam was subjected to bending and axial loads in the simply-supported condition. Figure 5 shows the response of the four strain sensors to bending loads. Similarly, Figure 6 shows the axial strain introduced to the temperature loading. For simulating complex loading, the beam was subjected to simultaneous bending and axial loads.

The sensors show good accuracy, precision and repeatability. There is a small level of noise in the measurements which makes the use of KF-based or similar approaches that are robust to measurement noise necessary. FBG 1 and and FBG 2 are symmetrically placed and as such show good agreement. This agreement is not seen in FBG 3 and FBG 4. A thorough investigation for the reason behind this discrepancy was carried out and reported in Ref. [27]. It was concluded that the reason behind this discrepancy was the imperfect sample preparation, which resulted in additional resin in the vicinity of FBG3. However, this discrepancy neither affects the linear nature of the strains measured nor the sensitivity of the sensors. As the KF-based methodology is based on the measured strains and their linear relationship alone, this bias does not affect the estimates of the strains. In reality, it points to the suitability of the method in applications with non-symmetric cross-section of the beams.

## 4. Numerical Results

### 4.1. Need for KF

**Figure 7:**
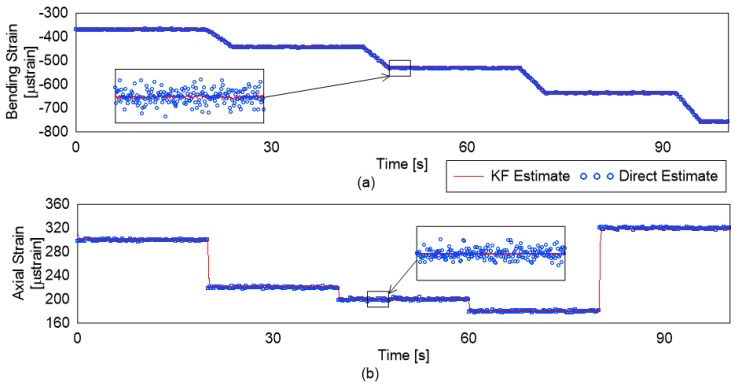
Strain estimation by DE- and KF-based estimation: (**a**) bending strains; and (**b**) axial strains.

The KF allows a weighted combination of the past estimates and the new estimate for the quantity of interest. As a result, under the presence of measurement noise (5% added noise in the simulation), it performs better than the direct estimation (DE) of the strains. This can be clearly observed in Figure 7. The DE is the estimate of the loads based on the measured strains at that instance calculated based on simultaneously solving Equations (1)–(3)。

For quantitative comparison, the mean and standard deviation of the bending strain estimate divided into periods of stable bending strain is shown in Table 1. Similar statistical analysis for axial strains is shown in Table 2.

It is evident from the results in the tables that the mean strain estimated by the KF is much closer to the real value than the DE. In addition, the standard deviation (std) is much lower for the KF based technique. This lower std is due to the more stable and accurate estimation. The values for the std for axial strain monitoring are higher than those for the bending strain for the KF based technique. This can be explained as the change in the bending strain being introduced gradually while the axial strain change being sudden in the simulation. The delay between the change in the strain value and the convergence may be adjusted based on the application requirements. It depends on the parameters of the R and Q matrix in the KF implementation. Based on the tables and the figure, it is obvious that the KF-based estimation brings accuracy and stability without much increase in the delay in the estimation of the true value.

### 4.2. Need for RNA Tracking

**Figure 8:**
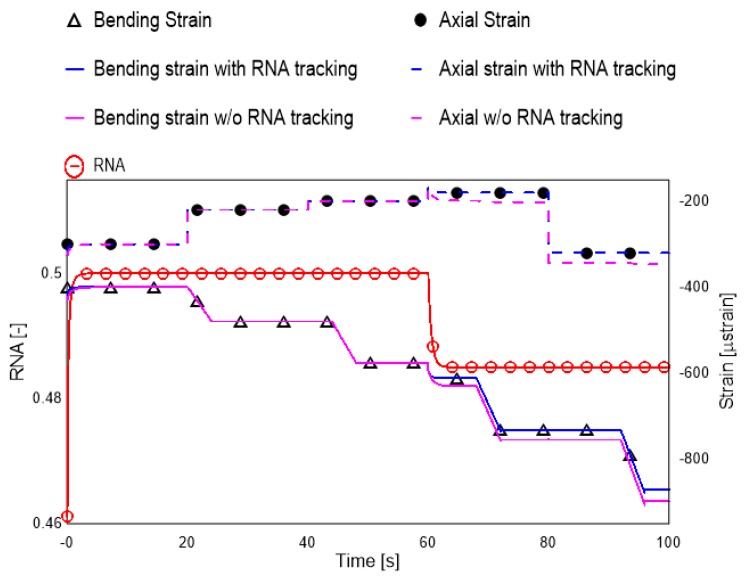
Need for RNA tracking in load estimation.

The RNA of the structure is a function of the condition of the structure alone and is independent of the applied loads. The strain measurements at RNA are insensitive to bending loads and this property is used for the estimation of the different strains. By achieving the RNA estimate as the part of the KF, errors in the estimation of the RNA, which may lead to wrong strain estimations, are overcome. A scenario was simulated where the RNA of the structure undergoes a sudden change. This is equivalent to development of sudden crack or delamination in the sample. The RNA, bending strains and the axial strain are shown in Figure 8.

It can be seen that, if the RNA is not tracked, an error (bias) is introduced in the axial and bending strain monitoring. The accurate load estimation is even more important in the case there is deterioration of the structure. This adds merit to the developed methodology. The experimental validation is presented in the next section.

## 5. Experimental Results

### 5.1. Load Monitoring

**Figure 9:**
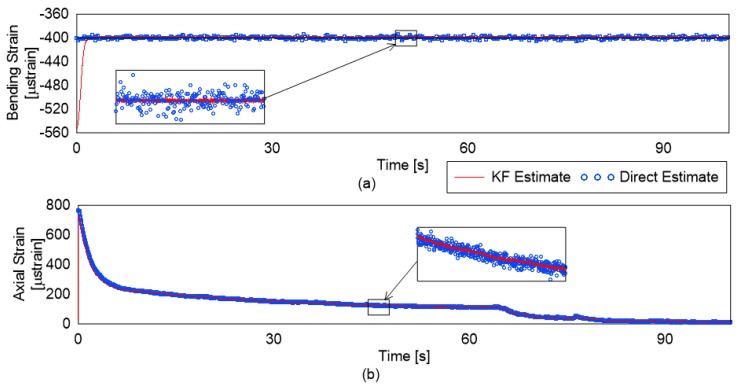
Load estimations under unloading case.

**Figure 10:**
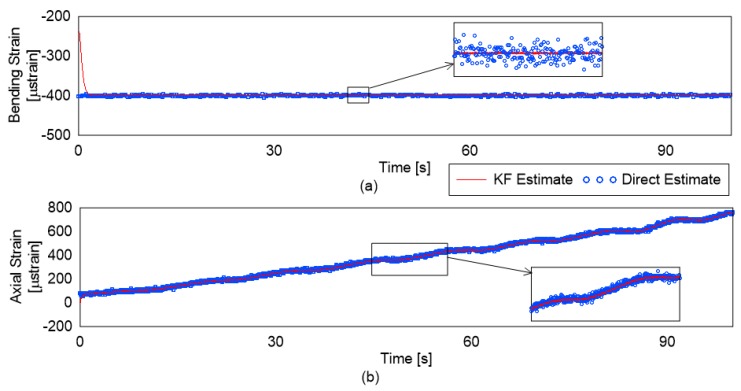
Load estimations under loading case.

Figure 9 and Figure 10 show the strain estimate for axial strains and bending strains in the unloading and loading scenarios, respectively. As can be seen, in the bending strains estimation in both cases, the loading was constant, as a result the expected trend is a straight line, which is seen once the convergence is achieved. The estimation based on the DE is not stable and has a much higher variance. For the axial strain, the loading is reducing until the equilibrium (baseline) conditions are reached for the unloading case. A point to note is that the axial strains were imposed through increase and decrease of temperature; as a result, the strain estimate is seen to follow the trend as predicted by Newton’s law of cooling. For the loading case, the agreement seen in the KF-based estimate is good, and the estimation is much more stable than that of DE.

### 5.2. Load Monitoring under Damage Scenarios

**Figure 11:**
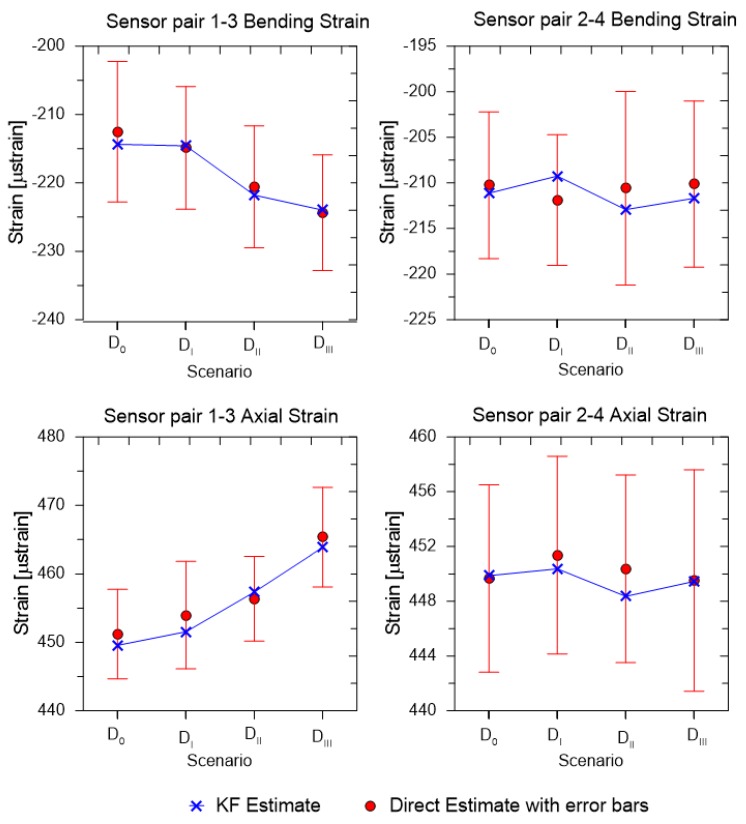
Load estimations for different damage scenarios.

As shown in the previous section, the tracking of RNA is essential for accurate estimation of strains, as the condition of the structure can affect the strain distribution. Thus, similar loading conditions were applied to the beam structure under four different scenarios. The details of the damage introduced may be found in Ref. [31]. Figure 11 shows the bending and axial strains under the different damage scenarios for the KF based estimation and DE. The DE is plotted with the error bars to show the errors in the estimation of DE due to the measurement noise. As can be seen, the strain distribution in sensor pair 2–4 is more or less constant (within reasonable measurement error) while the strains in the 1–3 sensor pair are higher as the level of damage increases. This shows that: (a) the tracking of RNA is required for accurate strain estimation; and (b) the KF based technique works better than the direct estimation. For the purpose of the DE based estimation, the RNA estimate was taken as a priori known, which is not possible. If the RNA estimation were not incorporated in the estimation, the estimates from the DE method would have a high error and bias, which would have been challenging to show in a scaled plot.

## 6. Conclusions and Future Work

This paper presents results from experiments as well as simulation studies on a validated FE model of a simple composite beam under changing axial loading conditions for the estimation of the bending and axial loads. The load estimation was carried out through the use of KF, which gives robustness in the presence of measurement noise as well as an opportunity to fuse different kinds of data. The KF is formulated with a parameter defined as the RNA, which is the true neutral axis of the beam. It allows accurate load estimation even when the beam experiences deterioration. This paper shows that the damage can be easily located using the RNA location as a damage sensitive feature. The implementation of this metric for real structures under operating conditions and fusing the temperature information as such is identified as the next step. The method aims to give a proof of concept of the formulation and the authors acknowledge that, train and track applications, where this method has the most potential for axial load monitoring, may pose some specific problems such as the impact effects of the approaching train or the rapid unloading when the axle passes the strain sensor. The validation under these conditions are identified as the next step. In addition, it is acknowledged that the method is for load estimation, yet the results are presented as the components of strain in bending and along the axis. The conversion of the strains to load is trivial in most cases, knowing the physical constants of the material and the cross-section. The paper does not present the results in the load form as the axial load was imposed through thermal loading and although equivalent to physical loading as such does not have any physical meaning.

## Figures and Tables

**Table 1:** Comparative performance of DE- and KF-based estimation for bending strains.

Time [s]	True Strain [μstrain]	KF Mean (std) [μstrain]	DE Mean (std) [μstrain]
0–20	369.2	369.2 (1.2)	369.2 (1.3)
24–44	443.0	443.0 (0.2)	440.1 (1.3)
48–68	531.7	531.7 (0.3)	534.6 (1.3)
72–92	636.9	636.9 (0.2)	631.9 (1.3)
96–100	756.9	756.9 (0.3)	752.5 (1.2)

**Table 2:** Comparative performance of DE- and KF-based estimation for axial strains.

Time [s]	True Strain [μstrain]	KF Mean (std) [μstrain]	DE Mean (std) [μstrain]
0–20	300.0	300.2 (0.8)	304.5 (1.3)
20–40	220.0	220.4 (0.4)	225.3 (1.3)
40–60	200.0	200.1 (0.6)	197.7 (1.2)
60–80	180.0	180.1 (0.5)	182.6 (1.3)
80–100	320.0	319.8 (0.6)	315.9 (1.2)
